# Impact of skeletal muscle mass on postoperative complications in oral cancer surgery

**DOI:** 10.1186/s40902-024-00417-w

**Published:** 2024-03-28

**Authors:** Arisa Fujii, Taiki Suzuki, Katsuhiko Sakai, Nobuyuki Matsuura, Keisuke Sugahara, Akira Katakura, Takeshi Nomura

**Affiliations:** 1 Department of Oral Pathobiological Science and Surgery, Tokyo Dental College, Tokyo, 101-0061 Japan; 2 Department of Oral Oncology, Oral and Maxillofacial Surgery, Tokyo Dental College, Chiba, 272-8513 Japan; 3 Department of Oral Medicine and Hospital Dentistry, Tokyo Dental College, Chiba, 272-8513 Japan

**Keywords:** Skeletal muscle mass, Postoperative adverse events, Oral cancer, Sarcopenia, Surgical site infection

## Abstract

**Background:**

Sarcopenia is characterized by a progressive and generalized loss of skeletal muscle mass and strength. The aim of this retrospective study was to investigate the impact of skeletal muscle mass on adverse events in free-flap reconstruction for defects after oral cancer resection.

**Results:**

Of 120 patients, recipient-site adverse events occurred in 56 patients (46.7%), and recipient-site surgical site infections occurred in 45 patients (37.5%). Skeletal muscle index was significantly associated with recipient-site adverse events in univariate analysis (*P* < 0.05). Lower body mass index and skeletal muscle index were significantly associated with recipient-site surgical site infection in univariate analysis (*P* < 0.05). In the multiple logistic regression model, a lower skeletal muscle index was a significant risk factor for recipient-site adverse events and surgical site infections (adverse events odds ratio; 3.17/*P* = 0.04; surgical site infection odds ratio; 3.76/*P* = 0.02).

**Conclusions:**

The SMI at level Th12 was an independent factor for postoperative AEs, especially SSI, in OSCC patients with free-flap reconstruction.

## Background

This study was performed to investigate the association between skeletal muscle mass and predictors of postoperative adverse events (AEs) in patients with oral squamous cell carcinoma (OSCC) who underwent free-flap reconstruction. Sarcopenia is a disease characterized by a progressive and generalized loss of skeletal muscle mass and strength, and a low skeletal muscle index (SMI) is a clinical assessment method of the disorder [1–3]. Previous studies have shown that a low SMI in patients with a variety of malignancies such as liver, lung, bladder, colorectal, and breast cancer, as well as head and neck cancer (HNC), results in more postoperative complications, more severe chemotherapy toxicity, and a lower survival rate than in those without a low SMI [3–12]. The incidence of AEs in patients undergoing free-flap reconstruction for HNC is higher and making early prevention and treatment important [13]. Moreover, prioritizing the early prevention and treatment of AEs is crucial as they influence the timing of initiating postoperative therapy. Predictors of AEs include age, sex, body mass index (BMI), performance status (PS), comorbidities, and recurrence; however, there is no standardized list.

Skeletal muscle measurement using computed tomography (CT) images at the L3 vertebral level has been widely used to evaluate low SMI in patients undergoing routine abdominal CT [14], but for patients with oral squamous cell carcinoma (OSCC), a limitation was posed in the applicability of assessing low SMI by evaluation at the L3 level. Swartz et al. found a strong correlation between the cross-sectional area (CSA) of muscles at the L3 and third cervical vertebra (C3) levels and reported that the assessment of skeletal muscle mass on head and neck CT is feasible [15]. Matsuyama et al. suggested that the assessment of the skeletal muscle area of the C3 level on CT in patients with HNC may be impaired by tumor invasion or lymphadenopathies and found a strong correlation between muscle CSA at L3 and that at the 12th thoracic spine (Th12) level [16]. No studies have evaluated the impact of muscle mass on patients with AEs using thoracic spine level images in OSCC. This study therefore aims to investigate such association by assessing the correlation between AEs and muscle mass assessed at the Th12 level in patients affected by OSCC.

## Methods

### Study setting and participants

This retrospective single-center cohort study was approved by the Ethical Review Committee of Tokyo Dental College Ichikawa General Hospital (approval ID: I 20–51). Clinical records were retrieved concerning 131 patients diagnosed with stages II–IV primary OSCC (according to the 7th edition of the American Joint Committee on Cancer [AJCC] staging manual) who underwent reconstructive surgery with a free flap (forearm flap, rectus abdominis muscle flap, anterolateral thigh flap, and fibular flap) at the Oral Cancer Center, Tokyo Dental College, between April 2014 and March 2019. Of these, those concerning two patients were excluded because their Th12 could not be measured, and data concerning further three patients were excluded because they did not undergo neck dissection. Therefore, clinical data assessed for this retrospective cohort study concerned 120 patients (85 men and 35 women).

### Data collection

Data collected from clinical records were age, sex, height, weight, BMI, tumor localization, TNM classification, SMI, American Society of Anesthesiologists physical status (ASA-PS) classification, smoking history, drinking status, the presence of diabetes mellitus, and preoperative laboratory data (hemoglobin, albumin, leukocytes, platelets, and C-reactive protein). Intraoperative factors, including the site of free flap harvest (anterolateral thigh, fibula, forearm, and others), operation time, blood loss, neck dissection method, and reconstruction method, were also recorded. Surgery-related complications were defined as those occurring within 30 days after the procedure, and all postoperative AEs were scored according to the Clavien–Dindo classification of surgical complications [17]. For each cut-off value, poor PS was defined as *ASA-PS* > 2 [18]. A BMI of < 18.5 kg/m^2^ was defined as underweight based on the definition of the Japan Society for the Study of Obesity (JASSO) [19].

### Image analysis

**Fig. 1:**
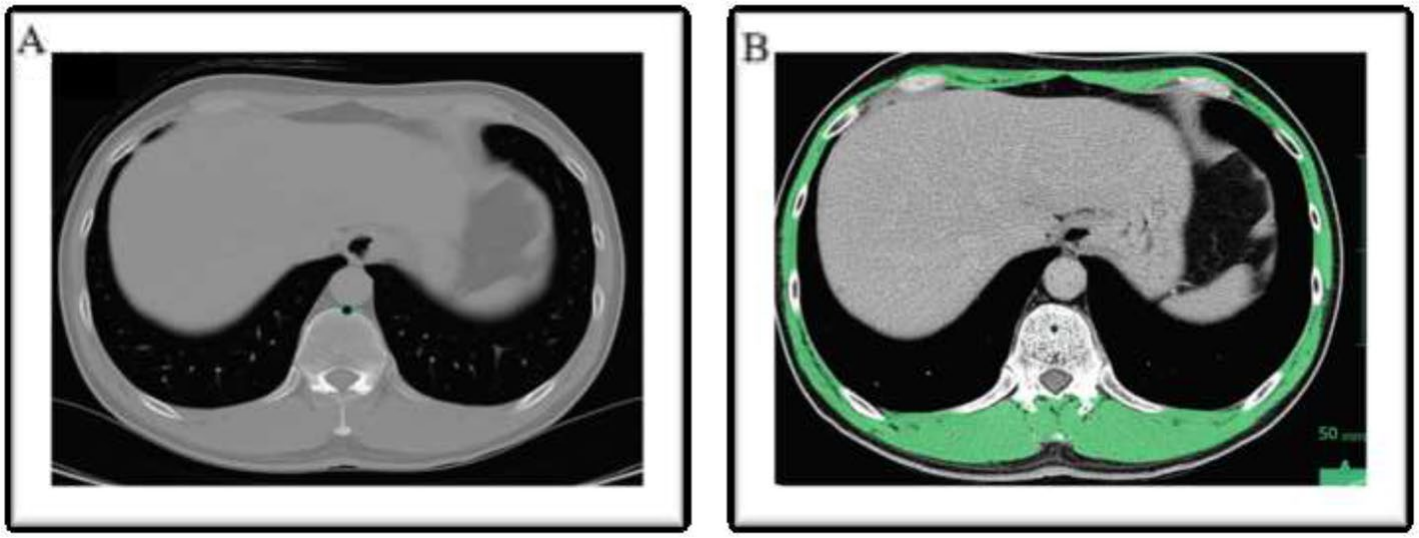
**a** The original CT image at Th12 transferred in the DICOM format. **b** The tissue HU threshold was set from − 29 to + 150 HU for skeletal muscle. The skeletal muscle was calculated by excluding the abdominal viscera and Th12

Axial CT images at the Th12 level of a cervicothoracic CT scan performed prior to the treatment for malignancy were used to measure the muscle CSA. The CSA measurement method used was that described by Matsuyama et al. [16]. CT images were scrolled from caudal to cephalic, and the Th12 CT slice with the clearest view of the costal process was selected [16]. Contrast agents (barium and iodine) commonly administered during normal CT scans have been reported to not interfere with the evaluation of skeletal muscle and abdominal adipose tissue [20]; thus, they were used to obtain contrast-enhanced CT images. The CSA of the muscles at Th12 was measured using the Vincent software (version 6, FUJIFILM Corp., Tokyo, Japan). One trained researcher (A. F.) performed all of the image analyses. CSA was measured semiautomatically and independently, and the values obtained from the two measurements were averaged and used in this study. For the measurement of skeletal muscle area, the magnification of the images, CT value threshold adjustment, and calculation of measurements were performed automatically by the software. The skeletal muscles were examined visually for anatomical features and manually traced. Skeletal muscles were identified using the standard Hounsfield unit in the range of − 29 to + 150 [16, 21]. The CSA of the delineated area was automatically calculated as the sum of the delineated pixels and was quantitatively evaluated. At the Th12 level slice, all muscles of the trunk wall, in addition to the erector muscles of the spine, were included (Fig. 1a, b). Subsequently, the skeletal muscle CSA at level L3 was estimated using the prediction rule described by Matsuyama et al. (Eq. (1)) [17]: the estimated skeletal muscle CSA at L3 was normalized for height by dividing it by the squared height: lumbar skeletal muscle index (L3SMI: lumbar 3 SMI, cm^2^/m^2^) (Eq. (2)). The optimal cut-off value for L3SMI was reported by Ohashi et al. [22] (men: 45.471 cm^2^/m^2^, women: 35.170 cm^2^/m^2^). This cut-off value correlated with the value from DXA, which is used to diagnose low skeletal muscle mass as defined by the Asian Working Group for Sarcopenia (AWGS) [23].

Equation 1 is as follows:

1 $$\mathrm{CSA\ at\ L}3\mathrm{\ level\ }({{\text{cm}}}^{2}) = 14.143 + 0.779 \times \mathrm{ CSA\ at\ Th}12\ ({{\text{cm}}}^{2})-0.212 \times \mathrm{ age\ }({\text{y}}) + 0.502 \times \mathrm{ weight\ }({\text{kg}}) + 13.763 \times \mathrm{ sex\ }({\text{women}}: 1,\mathrm{ men}: 2)$$

Equation 2 is as follows:

2 $${\text{L}}3\mathrm{SMI }=\mathrm{ L}3\mathrm{CSA }\times 1/(\mathrm{height }[{{\text{m}}}^{2}]).$$

### Statistical analysis

Data were summarized by means of descriptive statistics, that is by calculating the mean ± standard deviation (SD) or median (range) for continuous variables and reporting frequencies or calculating percentages (%) for categorical ones. As normality of continuous variables was rejected when they were assessed using the Kolmogorov–Smirnov test, differences in their distribution were investigated respectively by means of the Mann–Whitney test. Those of categorical ones were assessed using the *χ*^2^ test. To investigate the correlation between AEs and SMI, and to identify other independent risk factors associated with the occurrence of AEs in OSCC patients, data were assessed by means of univariate and multivariate analyses. The covariates used in the multivariate analysis were selected based on previous reports, so this study did not investigate the association between tumor stage and AEs. Multivariate analysis was performed using logistic regression analysis. All tests were two sided, and a *P*-value < 0.05 was considered statistically significant. All statistical analyses were performed using EZR software (64 bit) [24].

## Results

The characteristics of these 120 patients are presented in Table 1. The patients were aged 20–86 years, with a median age (interquartile range of 60 years; 85 were men, and 35 were women). According to the Union for International Cancer Control, the clinical stages were stage II in 15 cases, stage III in 15 cases, and stage IV in 90 cases. The primary tumor site was the tongue in 69 patients (57.5%), followed by the mandible in 26 (21.7%), floor of the mouth in 15 (12.5%), and buccal mucosa in 10 (8.3%).

**Table 1:** Patient characteristics

Characteristic	Number of patients (%)
Age (years), mean (range)	60 (20–86)
Sex	
Male	85 (70.8%)
Female	35 (29.2%)
BMI (kg/m^2^), mean ± SD	22.4 ± 4.0
Low BMI	
< 18.5	17 (14.2%)
≧ 18.5	103 (85.8%)
Stage	
II, III	30 (25.0%)
IV	90 (75.0%)
Location	
Tongue	69 (57.5%)
Mandible	26 (21.7%)
Oral floor	15 (12.5%)
Buccal mucosa	10 (8.30%)
Low SMI	20 (16.7%)
Neck dissection	
Unilateral	100 (83.3%)
Bilateral	20 (16.7%)

*BMI* body mass index, *SMI* skeletal muscle index

The mean values ± standard deviation of SMIs were 47.1 ± 8.1 (range: 28.0–69.1) cm^2^/m^2^ in all patients, 50.7 ± 6.4 cm^2^/m^2^ (range: 36.3–69.1) in men, and 38.9 ± 5.1 cm^2^/m^2^ (range: 28.0–57.3) in women.

AEs occurred in 57 (47.5%) patients. In univariate analysis, AEs were significantly associated with a lower SMI (Table 2). Multivariate analysis was conducted for factors considered to be associated with AEs, and lower SMI (odds ratio [OR] = 3.17, *P* = 0.04) and sex differences (*OR* = 2.45, *P* = 0.04) emerged as independent significant factors (Table 3). SSIs at the recipient site were observed in 45 patients (37.5%). In the univariate analysis, recipient-site SSI was significantly associated with lower BMI and SMI (Table 4). Multivariate analysis was performed for factors considered to be associated with SSI, and lower SMI (*OR* = 3.76, *P* = 0.02), sex difference (*OR* = 3.22, *P* = 0.01), and ACE27 (*OR* = 2.80, *P* = 0.03) emerged as independent significant factors (Table 5).

**Table 2:** Preoperative variables of patients who underwent oral cancer resection with flap reconstruction and did and did not have recipient-site surgical site infection (SSI)

Variable	Without AEs (*n* = 64)	With AEs (*n* = 56)	*p*-value
Age (years), median (range)	64 (30–86)	63 (26–80)	0.634a
Sex			
Male	50 (78.1%)	35 (62.5%)	0.072b
Female	14 (21.9%)	21 (37.5%)	
Low SMI	6 (9.4%)	14 (25.0%)	0.028b
BMI (kg/m^2^), mean ± SD	22.9 ± 4.1	21.8 ± 3.7	0.136a
Low BMI < 18.5	9 (14.1%)	8 (14.3%)	1.000b
Stage			
II, III	15 (23.4%)	15 (26.8%)	0.679b
IV	49 (76.6%)	41 (73.2%)	
Alcohol	35 (54.7%)	23 (41.1%)	0.707b
Smoking	33 (51.6%)	27 (48.2%)	0.855b
Location			
Tongue	40 (62.5%)	29 (51.8%)	0.150b
Mandible	9 (14.1%)	17 (30.4%)	
Buccal mucosa	5 (7.81%)	5 (8.93%)	
Floor of mouth	10 (15.6%)	5 (8.93%)	
Neck dissection			
Unilateral	53 (82.8%)	47 (83.9%)	1.00b
Bilateral	11 (17.2%)	9 (16.1%)	
ASA-PS score ≥ 2	27 (42.1%)	21 (37.5%)	0.709b
ACE-27			
0–1	26 (40.6%)	17 (30.4%)	0.259b
2–3	38 (59.4%)	39 (69.6%)	
Preoperative laboratory data, median (IQR)			
Leukocyte count (× 10^9^/l)	6.1 (5.2–8.0)	6.2 (5.0–7.5)	0.582a
Platelet count (× 10^9^/l)	235 (202–275)	240 (193–285)	0.807a
Hemoglobin (g/dl)	14.5 (14.1–14.8)	14.4 (14.0–14.7)	0.356a
Albumin (g/dl)	4.4 (4.1–4.6)	4.4 (4.1–5.3)	0.513a
C-reactive protein	0.09 (0.05–0.28)	0.075 (0.05–0.26)	0.475a
Prognostic nutritional index	51.3 (48.4–55.3)	52.6 (48.9–55.9)	0.566a
Operation time (min), median (IQR)	783 (688–873)	812 (668–857)	0.816a
Blood loss (ml), median (IQR)	670 (420–1027)	670 (450–950)	0.924a

*AE* adverse event, *ASA-PS* American Society of Anesthesiologists physical status, *BMI* body mass index, *IQR* interquartile range, *SMI* skeletal muscle index

**Table 3:** Significant risk factors for adverse events identified in multiple logistic regression analysis

Variable	OR	95%CI		*p*-value
		Lower	Upper	
Low SMI	3.17	1.08	9.31	0.04
Age	0.99	0.96	1.02	0.46
Sex	2.45	1.02	5.89	0.04
ACE-27	1.84	0.77	4.38	0.17
PNI	1.01	0.94	1.09	0.76
Blood loss (ml)	1.00	1.00	1.00	0.90

*CI* confidence interval, *OR* odds ratio, *PNI* prognostic nutritional index, *SMI* skeletal muscle index

**Table 4:** Perioperative variables of patients who underwent oral cancer resection with flap reconstruction and did and did not have recipient-site surgical site infection

Variable	Non-SSI (*n* = 75)	SSI (*n* = 45)	*p*-value
Age (years), median (range)	63 (23–86)	64 (20–80)	0.39a
Sex			
Male	58 (77.3%)	27 (60.0%)	0.06b
Female	17 (22.3%)	18 (40.0%)	
SMI (cm^2^/m^2^), mean ± SD	48.8 ± 7.4	44.2 ± 8.6	0.002a
Low SMI	7 (9.3%)	13 (28.9%)	0.001b
BMI (kg/m^2^), mean ± SD	23.0 ± 4.1	21.3 ± 3.6	0.020a
Low BMI < 18.5	10 (13.3%)	7 (15.5%)	0.793b
Stage			
II, III	25 (33.3%)	10 (22.2%)	0.667b
IV	55 (73.3%)	35 (77.8%)	
Alcohol	30 (40%)	28 (62.2%)	0.855b
Smoking	36 (48%)	24 (44.4%)	0.855b
Location			
Tongue	47 (62.7%)	22 (48.9%)	0.328b
Mandible	13 (17.3%)	13 (28.9%)	
Buccal mucosa	5 (6.7%)	5 (11.1%)	
Floor of mouth	10 (13.3%)	5 (11.1%)	
Neck dissection			
Unilateral	64 (85.3%)	36 (80.0%)	0.459b
Bilateral	11 (14.7%)	9 (20.0%)	
ASA-PS score ≧ 2	27 (45.8%)	23 (35.4%)	0.274b
ACE-27			
0–1	30 (40.0%)	13 (28.9%)	0.244b
2–3	45 (60.0%)	32 (71.1%)	
Preoperative laboratory data, median (IQR)			
Leukocyte count (× 10^9^/l)	6.2 (5.3–8.2)	6.2 (4.9–6.8)	0.205a
Platelet count (× 10^9^/l)	23.3 (20.1–27.6)	24.2 (20.6–28.2)	0.591a
Hemoglobin (g/dl)	14.5 (14.1–14.8)	14.3 (13.9–14.7)	0.100a
Albumin (g/dl)	4.4 (4.1–4.7)	4.4 (4.1–5.3)	0.746a
Prognostic nutritional index	52.1 (49.0–55.8)	52.0 (48.4–54.5)	0.408a
Operation time (min), median (IQR)	783 (688–873)	812 (668–857)	0.816a
Blood loss (ml), median (IQR)	670 (420–1027)	670 (450–950)	0.924a

*ASA-PS* American Society of Anesthesiologists physical status, *BMI* body mass index, *IQR* interquartile range, *SMI* skeletal muscle index

**Table 5:** Significant risk factors for recipient-site surgical site infection identified using multiple logistic regression analysis

Variable	OR	95% *CI*		*p*-value
		Lower	Upper	
Low SMI	3.76	1.27	11.10	0.02
Age	1.00	0.97	1.04	0.87
Sex	3.22	1.27	8.17	0.01
ACE-27	2.80	1.10	7.10	0.03
PNI	0.96	0.89	1.04	0.36

*CI* confidence interval, *OR* odds ratio, *PNI* prognostic nutritional index, *SMI* skeletal muscle index

## Discussion

There are currently no reports examining muscle mass using thoracic spine level images in patients with OSCC, and, to our knowledge, this is the first study to demonstrate the association of AEs and SSIs with Th12 SMI.

Although the evaluation of skeletal muscle area at the C3 level on CT in patients with OSCC is common, in our study, there were many inter-rater errors, and more patients were excluded than in the Th12 measurement group owing to the influence of the imaging axis.

SSI is defined by the Centers for Disease Control and Prevention as a wound infection that occurs within 30 days of an operative procedure. The incidence of postoperative AE, especially SSI, is high after oral cancer surgery because patients probably have poor nutrition prior to surgery, a dead space is likely to form anatomically, and the wound is exposed to saliva and fluid retained in the oral cavity [18, 25–32]. Therefore, it is important to control the risk of AEs before surgery.

To date, hypoalbuminemia, long surgical time, and high ASA scores have been reported as risk factors for SSI after oral cancer surgery [15, 25–32]. In this study, a decrease in muscle mass, which is closely related to a low SMI, was shown to be a risk factor for SSI after oral cancer surgery. However, no significant associations were observed for hypoalbuminemia and long surgical time. The reason for this is that the The European Society of Parenteral and Enteral Nutrition guidelines recommend postponing surgery if the serum albumin level is less than 3.0 g/dl [33]. In the present study, there was only one patient with a serum albumin level of less than 3.0 g/dl, which was presumably insufficient for a statistical study. In addition, the surgical time suggested to be associated with AEs (especially flap complications) is > 10 h [34]. In this study, flap necrosis occurred in 16 patients, and the mean operative time was 12 h 31 min. The mean operative time for patients without necrosis was 16 h 22 min. In a study on lung cancer, *PS* ≥ 2 was associated with poor overall survival [35, 36]. Similarly, in our study, PS was a predictor of prognosis, which is a reasonable finding.

The results of this study suggest that the female sex could be a risk factor for SSI. Recently, skeletal muscle was identified as an organ that secretes myokines that may influence the growth of cancer cells [37]. Hojman et al. reported that myokines, which are released from muscles during exercise, inhibit the growth of breast cancer cells and induce apoptosis of these cells [38]. Therefore, as muscle mass decreases, myokine levels and responses may also decrease, making it easier for cancer to progress.

In our study, univariate analysis results demonstrated that BMI was not statistically significantly different from postoperative AEs. Notably, conventional nutritional assessments do not reliably detect skeletal muscle loss [39]. BMI alone makes it difficult to distinguish between fat and fat-free mass [40], presumably because weight loss cannot differentiate between adipose tissue and muscle loss.

This study has some limitations. As it was conducted at a single center, the presence of an unmeasured bias cannot be ruled out. According to the Asian Working Group for Sarcopenia (AWGS) guidelines, the diagnosis of low SMI requires low muscle strength or low physical performance in addition to low muscle mass. The present study did not consider muscle strength, as its measurements were not available in the clinical records being assessed. A further limitation is operative in nature: as the the AWGS has not proposed clear SMI cut-off values for Th12, the one used in the present study is not standardized, as in previous studies concerning SMI. However, such skeletal muscle mass measurement might still be an additional useful predictor of postoperative AEs, especially when used in conjunction with those already assessed in the clinical setting. Other limitations of the present study include the selection of covariates — not including, for example, the tumor stage, as well as those stemming from it being retrospective. To address these limitations, it is suggested that future studies employ more comprehensive analyses that include more covariates and a prospective study design. In addition, larger validation studies and clinical trials with different patient populations should be conducted to confirm preliminary findings. This would further solidify the results of this study and confirm the utility of the proposed predictors.

## Conclusions

The SMI at the Th12 level was an independent factor for postoperative AEs, especially SSI, in OSCC patients with free-flap reconstruction, suggesting this might be a useful predictive factor for AEs in the clinical setting. Such results though are preliminary, and prospective studies on a greater number of subjects should be warranted to further investigate such clinically relevant matter.

## Data Availability

The datasets used and/or analyzed during the current study are available from the corresponding author on reasonable request.

## Abbreviations

AE: Adverse events
AJCC: American Joint Committee on Cancer
ASA-PS: American Society of Anesthesiologists physical status
AWGS: Asian Working Group for Sarcopenia
BMI: Body mass index
CSA: Cross-sectional area
CT: Computed tomography
DXA: Dual-energy X-ray absorptiometry
HNC: Head and neck cancer
OR: Odds ratio
OSCC: Oral squamous cell carcinoma
PNI: Prognostic nutritional index
PS: Performance status
SD: Standard deviation
SMI: Skeletal muscle index
SSI: Surgical site infection

## References

[1] Rosenberg IH (1989). Summary comments. Am J Clin Nutr.

[2] Cruz-Jentoft AJ, Baeyens JP, Bauer JM, Boirie Y, Cederholm T, Landi F (2010). Sarcopenia: European consensus on definition and diagnosis: report of the European Working Group on Sarcopenia in older people. Age Ageing.

[3] Chindapasirt J (2016). Sarcopenia in cancer patients. Asian Pac J Cancer Prev.

[4] Harimoto N, Shirabe K, Yamashita YI, Ikegami T, Yoshizumi T, Soejima Y (2013). Sarcopenia as a predictor of prognosis in patients following hepatectomy for hepatocellular carcinoma. Br J Surg.

[5] Barret M, Antoun S, Dalban C, Malka D, Mansourbakht T, Zaanan A (2014). Sarcopenia is linked to treatment toxicity in patients with metastatic colorectal cancer. Nutr Cancer.

[6] Otsuji H, Yokoyama Y, Ebata T, Igami T, Sugawara G, Mizuno T (2015). Preoperative sarcopenia negatively impacts postoperative outcomes following major hepatectomy with extrahepatic bile duct resection. World J Surg.

[7] Jung HW, Kim JW, Kim JY, Kim SW, Yang HK, Lee JW (2015). Effect of muscle mass on toxicity and survival in patients with colon cancer undergoing adjuvant chemotherapy. Support Care Cancer.

[8] Chen LK, Liu LK, Woo J, Assantachai P, Auyeung TW, Bahyah KS (2014). Sarcopenia in Asia: consensus report of the Asian Working Group for Sarcopenia. J Am Med Dir.

[9] Wendrich AW, Swartz JE, Bril SI, Wegner I, de Graeff A, Smid EJ (2017). Low skeletal muscle mass is a predictive factor for chemotherapy dose-limiting toxicity in patients with locally advanced head and neck cancer. Oral Oncol.

[10] Cho Y, Kim JW, Keum KC, Lee CG, Jeung HC, Lee IJ (2018). Prognostic significance of sarcopenia with inflammation in patients with head and neck cancer who underwent definitive chemoradiotherapy. Front Oncol.

[11] Fattouh M, Chang GY, Ow TJ, Shifteh K, Rosenblatt G, Patel VM (2019). Association between pretreatment obesity, sarcopenia, and survival in patients with head and neck cancer. Head Neck.

[12] Nakamura H, Makiguchi T, Yamaguchi T, Suzuki K, Yokoo S (2020). Impact of sarcopenia on postoperative surgical site infections in patients undergoing flap reconstruction for oral cancer. Int J Oral Maxillofac Surg.

[13] Patel RS, McCluskey SA, Goldstein DP, Minkovich L, Irish JC, Brown DH (2010). Clinicopathologic and therapeutic risk factors for perioperative complications and prolonged hospital stay in free flap reconstruction of the head and neck. Head Neck.

[14] Brown JC, Cespedes Feliciano EM, Caan BJ (2018). The evolution of body composition in oncology—epidemiology, clinical trials, and the future of patient care: facts and numbers. J Cachexia Sarcopenia.

[15] Swartz JE, Pothen AJ, Wegner I, Smid EJ, Swart KM, de Bree R (2016). Feasibility of using head and neck CT imaging to assess skeletal muscle mass in head and neck cancer patients. Oral Oncol.

[16] Matsuyama R, Maeda K, Yamanaka Y, Ishida Y, Kato R, Nonogaki T (2021). Assessing skeletal muscle mass based on the cross-sectional area of muscles at the 12th thoracic vertebra level on computed tomography in patients with oral squamous cell carcinoma. Oral Oncol.

[17] Dindo D, Demartines N, Clavien PA (2004). Classification of surgical complications: a new proposal with evaluation in a cohort of 6336 patients and results of a survey. Ann Surg.

[18] Kamizono K, Sakuraba M, Nagamatsu S, Miyamoto S, Hayashi R (2014). Statistical analysis of surgical site infection after head and neck reconstructive surgery. Ann Surg Oncol.

[19] Kanazawa M, Yoshiike N, Osaka T, Numba Y, Zimmet P, Inoue S (2002). Criteria and classification of obesity in Japan and Asia-Oceania. World Rev Nutr Diet.

[20] Mitsiopoulos N, Baumgartner RN, Heymsfield SB, Lyons W, Gallagher D, Ross R (1998). Cadaver validation of skeletal muscle measurement by magnetic resonance imaging and computerized tomography. J Appl Physiol.

[21] Nemec U, Heidinger B, Sokas C, Chu L, Eisenberg RL (2017). Diagnosing sarcopenia on thoracic computed tomography: quantitative assessment of skeletal muscle mass in patients undergoing transcatheter aortic valve replacement. Acad Radiol.

[22] Ohashi K, Ishikawa T, Hoshii A, Hokari T, Noguchi H, Suzuki M (2021). Optimal skeletal muscle mass index cut-off values for presarcopenia evaluated by computed tomography against dual-energy x-ray absorptiometry in patients with chronic liver disease. J Clin Med.

[23] Chen LK, Lee WJ, Peng LN, Liu LK, Arai H, Akishita M (2016). Recent advances in sarcopenia research in Asia: 2016 update from the Asian Working Group for Sarcopenia. J Am Med Dir Assoc.

[24] Kanda Y (2013). Investigation of the freely available easy-to-use software 'EZR' for medical statistics. Bone Marrow Transplant.

[25] Karakida K, Aoki T, Ota Y, Yamazaki H, Otsuru M, Takahashi M (2010). Analysis of risk factors for surgical-site infections in 276 oral cancer surgeries with microvascular free-flap reconstructions at a single university hospital. J Infect Chemother.

[26] Lee DH, Kim SY, Nam SY, Choi SH, Choi JW, Roh JL (2011). Risk factors of surgical site infection in patients undergoing major oncological surgery for head and neck cancer. Oral Oncol.

[27] Belusic-Gobic M, Car M, Juretic M, Cerovic R, Gobic D, Golubovic V (2007). Risk factors for wound infection after oral cancer surgery. Oral Oncol.

[28] Goyal N, Yarlagadda BB, Deschler DG, Emerick KS, Lin DT, Rich DL (2017). Surgical site infections in major head and neck surgeries involving pedicled flap reconstruction. Ann Otol Rhinol.

[29] Liu SA, Wong YK, Poon CK, Wang CC, Wang CP, Tung KC (2007). Risk factors for wound infection after surgery in primary oral cavity cancer patients. Laryngoscope.

[30] Ogihara H, Takeuchi K, Majima Y (2009). Risk factors of postoperative infection in head and neck surgery. Auris Nasus Larynx.

[31] Yang CH, Chew KY, Solomkin JS, Lin PY, Chiang YC, Kuo YR (2013). Surgical site infections among high-risk patients in clean-contaminated head and neck reconstructive surgery: concordance with preoperative oral flora. Ann Plast Surg.

[32] Hirakawa H, Hasegawa Y, Hanai N, Ozawa T, Hyodo I, Suzuki M (2013). Surgical site infection in clean-contaminated head and neck cancer surgery: risk factors and prognosis. Eur Arch Otorhinolaryngol.

[33] Braga M, Ljungqvist O, Soeters P, Fearon K, Weimann A, Bozzetti F (2009). ESPEN guidelines on parenteral nutrition: surgery. Clin Nutr.

[34] Singh B, Cordeiro PG, Santamaria E, Shaha AR, Pfister DG, Shah JP (1999). Factors associated with complications in microvascular reconstruction of head and neck defects. Plast Reconstr Surg.

[35] Bowden JCS, Williams LJ, Simms A, Price A, Campbell S, Fallon MT (2017). Prediction of 90 day and overall survival after chemoradiotherapy for lung cancer: role of performance status and body composition. Clin Oncol (R Coll Radiol).

[36] Katsui K, Ogata T, Sugiyama S, Yoshio K, Kuroda M, Hiraki T (2021). Sarcopenia is associated with poor prognosis after chemoradiotherapy in patients with stage III non-small-cell lung cancer: a retrospective analysis. Sci Rep.

[37] Pedersen BK, Febbraio MA (2012). Muscles, exercise and obesity: skeletal muscle as a secretory organ. Nat Rev Endocrinol.

[38] Hojman P, Dethlefsen C, Brandt C, Hansen J, Pedersen L, Pedersen BK (2011). Exercise-induced muscle-derived cytokines inhibit mammary cancer cell growth. Am J Physiol Endocrinol Metab.

[39] Lou N, Chi CH, Chen XD, Zhou CJ, Wang SL, Zhuang CL (2017). Sarcopenia in overweight and obese patients is a predictive factor for postoperative complication in gastric cancer: a prospective study. Eur J Surg Oncol.

[40] Janssen I, Mark AE (2007). Elevated body mass index and mortality risk in the elderly. Obes Rev.

